# Adsorptive Removal of Pharmaceuticals and Personal Care Products from Water with Functionalized Metal-organic Frameworks: Remarkable Adsorbents with Hydrogen-bonding Abilities

**DOI:** 10.1038/srep34462

**Published:** 2016-10-03

**Authors:** Pill Won Seo, Biswa Nath Bhadra, Imteaz Ahmed, Nazmul Abedin Khan, Sung Hwa Jhung

**Affiliations:** 1 Department of Chemistry, Kyungpook National University, Daegu 702-701, Korea

## Abstract

Adsorption of typical pharmaceuticals and personal care products (PPCPs) (such as naproxen, ibuprofen and oxybenzone) from aqueous solutions was studied by using the highly porous metal-organic framework (MOF) MIL-101 with and without functionalization. Adsorption results showed that MIL-101s with H-donor functional groups such as –OH and –NH_2_ were very effective for naproxen adsorption, despite a decrease in porosity, probably because of H-bonding between O atoms on naproxen and H atoms on the adsorbent. For this reason, MIL-101 with two functional groups capable of H-bonding (MIL-101-(OH)_2_) exhibited remarkable adsorption capacity based on adsorbent surface area. The favorable contributions of –OH and –(OH)_2_ on MIL-101 in the increased adsorption of ibuprofen and oxybenzone (especially based on porosity) confirmed again the importance of H-bonding mechanism. The adsorbent with the highest adsorption capacity, MIL-101-OH, was very competitive when compared with carbonaceous materials, mesoporous materials, and pristine MIL-101. Moreover, the MIL-101-OH could be recycled several times by simply washing with ethanol, suggesting potential application in the adsorptive removal of PPCPs from water.

Recently, pharmaceuticals and personal care products (PPCPs) have attracted much attention because of their necessity in everyday life and huge production/consumption worldwide^1^,^2^,^3^,^4^,^5^,^6^. PPCPs may often remain in the environment even after they have been consumed completely^1^,^2^,^3^,^4^,^5^,^6^ because PPCPs usually have long shelf lives to meet customers’ demands, and some PPCPs are inadvertently dumped into the environment. For example, various PPCPs have recently been found in surface water, ground water, and even in the tissues of fishes and vegetables^1^,^2^,^3^,^4^,^5^,^6^,^7^,^8^,^9^,^10^; therefore, PPCPs are typical examples of so-called emerging contaminants^5^,^6^. It is reported that PPCPs may cause endocrine disruptions that can change hormonal actions^1^,^2^,^3^,^4^,^5^,^6^,^7^,^8^,^9^,^10^, although the adverse impact of PPCPs on human health and the environment is still not fully understood. Therefore, the removal of these PPCPs from surface/ground water and aquatic systems has recently been attracting much attention^1^,^2^,^3^,^4^,^5^,^6^,^7^,^8^,^9^,^10^,^11^,^12^ even though PPCPs in the environment have not been regulated explicitly so far.

Several methods, including biodegradation, chlorination, and advanced oxidation processes (AOPs)/ozonation, have been applied for the removal of PPCPs^1^,^2^,^3^,^4^,^5^,^6^,^7^,^8^,^9^,^10^,^11^,^12^,^13^,^14^; however, removal of PPCPs from water has not proven very successful and requires further improvement. For example, AOP and ozonation have the disadvantages of high energy consumption and the formation of residual byproducts^13^,^14^, respectively. Adsorption is a potential method for the removal of PPCPs considering the mild operation conditions, low energy consumption, and lack of side products. So far, carbonaceous materials (including activated carbon, carbon nanotubes, and bone char)^3^,^15^ and mesoporous materials (transition metal-grafted)^11^,^12^ have been widely studied as potential adsorbents for the removal of PPCPs.

There has been remarkable progress in research on nanoporous materials such as metal-organic frameworks (MOFs)^16^,^17^,^18^,^19^,^20^,^21^,^22^,^23^,^24^ and mesoporous materials^25^,^26^,^27^ in terms of both synthesis and applications. MOFs are composed of both metallic and organic species, and can have huge porosity in the microporous or mesoporous range; therefore, they have attracted much attention. Importantly, MOFs can be modified easily for various purposes by functionalization using coordinatively unsaturated sites (CUSs)^28^ or organic linkers. Several virgin and functionalized MOFs have been used for the adsorptive removal of hazardous materials^19^,^20^,^21^,^29^,^30^,^31^,^32^ based on various interaction mechanisms^29^,^30^,^31^,^32^ such as simple electrostatic interactions, acid-base interactions, coordination, and so on. Purification of fuels via adsorptive desulfurization and denitrogenation is one of the most deeply studied applications of MOFs^33^,^34^,^35^,^36^,^37^,^38^,^39^,^40^. Water purification is another important field of application of MOFs^41^,^42^,^43^,^44^; however, functionalization of MOFs is expected to be important in water purification because MOFs are usually hydrophilic and ineffective for water purification without incorporating functionalities with special interactions^45^.

Recently, we have reported the potential application of MOFs such as MIL-101 and MIL-100 to the adsorptive removal of PPCPs with or without functionalization with acidic or basic sites^46^,^47^. We suggested that adsorptive removal could occur via electrostatic and acid-base interactions. Herein, we report that functionalized MOFs, particularly those with free hydroxyl groups, can be effectively utilized for adsorptive removal of PPCPs, likely because of contributions from H-bonding. In this study, we used MIL-101 and naproxen as representatives MOF and PPCP, respectively. Adsorption of other PPCPs such as ibuprofen and oxybenzone were also carried out to understand the adsorption more.

MIL-101, Cr_3_O(F/OH)(H_2_O)_2_[C_6_H_4_(CO_2_)_2_], is one of the most widely studied MOFs with huge porosity and wide pore sizes^48^. MIL-101 has CUSs suitable for modification^28^, and therefore it can be used for various applications such as adsorption^49^ and catalysis^50^ after modification. Naproxen and ibuprofen are nonsteroidal anti-inflammatory drugs that are widely used to reduce pain, inflammation, fever, and stiffness. Oxybenzone is a component of many sunscreen lotions. Naproxen, ibuprofen and oxybenzone are regarded as typical emerging contaminants with high environmental risk. The chemical structures of naproxen, ibuprofen and oxybenzone are shown in Fig. 1. The three PPCPs have various functional groups such as free carboxylic acid, phenol, ketone and ether groups that can interact effectively with adsorbents such as functionalized MOFs. The physical properties of the PPCPs are summarized in Supplementary Table 1.

## Results

### Characteristics of the adsorbents

**Figure 2:**
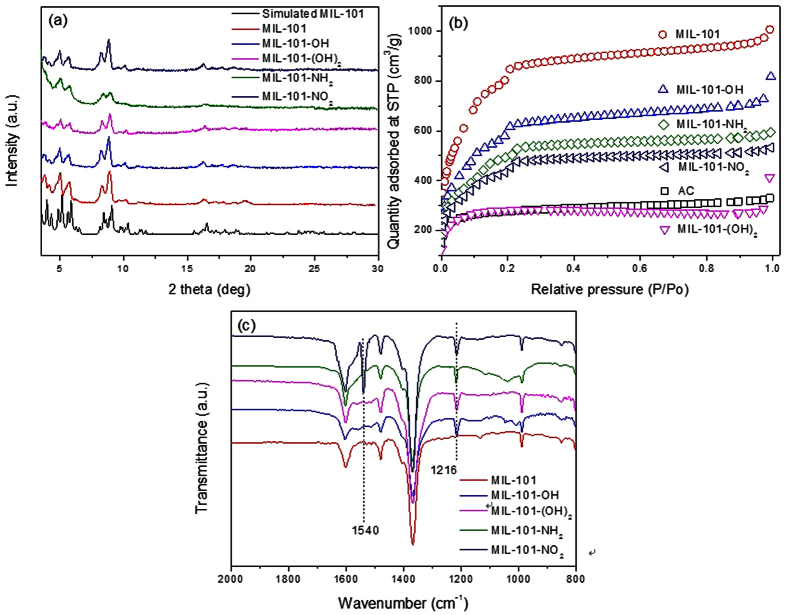
(**a**) XRD patterns, (**b**) nitrogen adsorption isotherms and (**c**) FTIR spectra of MIL-101s.

The XRD patterns of the MIL-101s shown in Fig. 2a are agreeable with simulated one^48^,^51^, confirming the MIL-101s were successfully prepared and that the crystal structure of pristine MIL-101 does not change with functionalization. However, the XRD intensities of the MIL-101s decreased slightly on modification, particularly those of MIL-101-(OH)_2_ and MIL-101-NH_2_, probably because of harsh conditions required for these modifications. The nitrogen adsorption isotherms (Fig. 2b) of the MIL-101s and the BET surface areas (Table 1) obtained from these isotherms show that the MIL-101s have considerable porosities, although functionalization (to introduce –OH, –NO_2_, and –NH_2_ groups) reduced the porosities. This reduction could be due to the volumes of the functional groups and/or the decreased crystallinity with modifications (as shown by the XRD patterns). FTIR spectra of the modified MOFs shown in Fig. 2c confirm the grafting was successful based on the presence of the band at 1216 cm^−1^, which originate from the C-N stretching of the grafting agents^52^. The band at 1540 cm^−1^ of the MIL-101-NO_2_ is because of the stretching vibration of -NO_2_ group^53^.

### Comparison of adsorbents for naproxen adsorption

**Figure 3:**
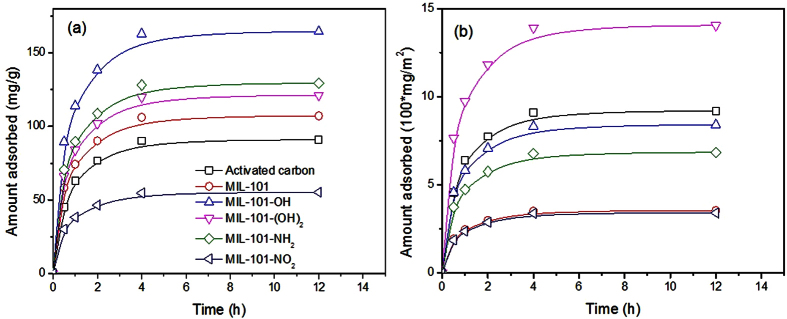
Effect of adsorption times on the adsorbed amounts of naproxen over MIL-101s and activated carbon. (**a**,**b**) Show the adsorbed amounts of naproxen based on the unit weight and surface area, respectively, of adsorbents. The initial concentration of naproxen was 50 ppm. The legends in (**b**) are the very same as those in (**a**).

Figure 3 shows the quantity of naproxen adsorbed by MIL-101s (based on weight and surface area of adsorbents) and activated carbon at different adsorption times. The figure indicates that naproxen adsorption by MIL-101s and activated carbon was almost complete after 4 h, suggesting relatively rapid adsorption of this material. As illustrated in Fig. 3a, the amount adsorbed (based on weight of adsorbents) decreased in the order MIL-101–OH > MIL-101-NH_2_ > MIL-101-(OH)_2_ > MIL-101 > activated carbon > MIL-101-NO_2_, which agrees with reported results for virgin MIL-101, MIL-101-NH_2_, and activated carbon^46^,^47^. Although the surface area of MIL-101-OH was around 70% that of pristine MIL-101, it adsorbed much more (about 1.53 times after 12 h) naproxen. Moreover, the results show the very high competitiveness (about 1.81 times after 12 h) of the MOFs against conventional adsorbents such as activated carbon. Figure 3b shows the amount of naproxen adsorbed per unit surface area by the MIL-101s and activated carbon, which decreased in the order MIL-101-(OH)_2_ > activated carbon > MIL-101-OH > MIL-101-NH_2_ > MIL-101 ~ MIL-101-NO_2_. The MIL-101-(OH)_2_ showed very high adsorption capacity for naproxen per unit surface area, even though the amount of naproxen adsorbed per unit weight was not very high. Curiously, however, MIL-101-NO_2_ was very poor at naproxen adsorption based on both weight and surface area despite the presence of the polar nitro group in the MOF.

### Adsorption isotherms and effect of functional groups on adsorption

**Figure 4:**
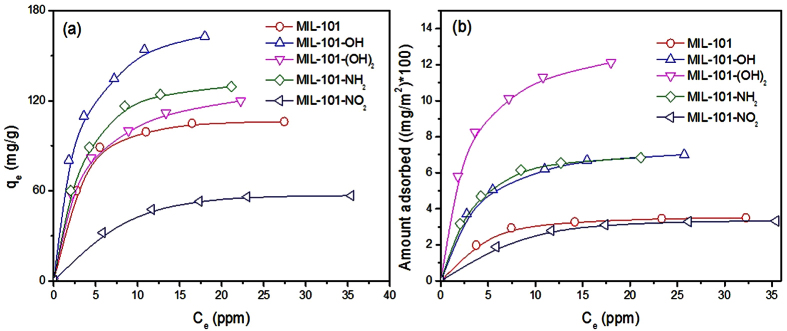
Adsorption isotherms of naproxen over MIL-101s at 25 °C. (**a**,**b**) Show the isotherms of naproxen based on the unit weight and surface area, respectively, of adsorbents.

Isotherms for naproxen adsorption by MIL-101s were obtained at 25 °C after 12 h of adsorption, which is sufficient for equilibrium, and the results are shown in Fig. 4. The adsorbed amounts (based on weight of MIL-101s) at equilibrium decreased in the order MIL-101–OH > MIL-101-NH_2_ > MIL-101-(OH)_2_ > MIL-101 > MIL-101-NO_2_, which was the same order as observed for quantity adsorbed after various times (Fig. 3a). The adsorbed amounts per unit area decreased in the order MIL-101–(OH)_2_ > MIL-101-OH ~ MIL-101-NH_2_ > MIL-101 ~ MIL-101-NO_2_, in agreement with Fig. 3b. The maximum adsorbed quantities (*Q*_*0*_) obtained from Langmuir plots (Supplementary Figure 1) are summarized in Table 1, and the results again show that MIL-101s functionalized with –OH groups were highly effective at adsorbing naproxen from water. The amino group was also effective at naproxen adsorption, in agreement with a previous study^47^ despite the use of a different functionalization method. However, as shown in Figs 3 and 4, the introduction of a nitro group on the surface of MIL-101 was not effective for the adsorption of naproxen, even with the presence of charge separations in the –NO_2_ group (positive N and negative O). Very curiously, the MIL-101-NO_2_ and pristine MIL-101 showed very similar performances (based on surface area) for naproxen adsorption as shown in Figs 3b and 4b. Considering the functional groups on naproxen, including a carboxylic acid and an ether, the presence of polar groups on MIL-101s was expected to yield effective adsorption of naproxen via, for example, electrostatic interactions^54^; however, only –OH and –NH_2_ groups were efficient for absorption of naproxen from water.

### Effect of solution pH

**Figure 5:**
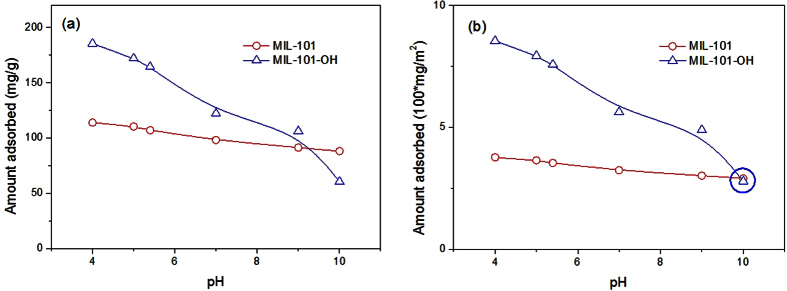
Effect of pH of solution on the adsorbed amounts of naproxen over MIL-101 and MIL-101-OH. (**a**,**b**) Show adsorbed amounts based on the unit weight and surface area, respectively, of adsorbents.

The pH of a solution is very important^54^ in the adsorption of organics from water considering the protonation/deprotonation of adsorbates and/or changes in the surface charges of adsorbents with different pH values. In this work, MOFs such as MIL-101-OH and pristine MIL-101 were studied at various pH values. As shown in Fig. 5, the amounts of naproxen adsorbed by MIL-101 and MIL-101-OH decreased as solution pH increased, which is similar to previous results for pristine MIL-101^46^,^47^. This tendency is understandable considering the ready deprotonation of naproxen at high pH (pKa of naproxen ~4.2) and the decreased surface charge (i.e., a change from positive to negative) of MIL-101^55^ with increasing pH. In other words, repulsive interactions between MIL-101s and naproxen are expected at high pH. Very curiously, the amount adsorbed by MIL-101-OH per unit surface area at a pH 10 was very similar to that of pristine MIL-101 (highlighted with a blue circle in Fig. 5b). This could be due to deprotonation of the –OH group (to form –O^−^) in MIL-101-OH at pH 10 (considering the pKa of ethanolamine, 9.5), leading to the contribution of H-bonding between naproxen and deprotonated MIL-101-OH becoming negligible, meaning only surface area was important in adsorption (see below).

### Competitiveness and reusability of the adsorbent

So far, several adsorbents including carbonaceous materials have been used to adsorb naproxen from water. Table 2 compares the maximum adsorption capacities (*Q*_*0*_) and amounts adsorbed at equilibrium (*q*_*24 h*_, after 24 h) of studied adsorbents. Table 2 shows that the MIL-101-OH was very competitive when compared to studied adsorbents such as activated carbon^11^,^56^,^57^,^58^, bone char^15^, and mesoporous materials with and without modifications (SBA-15^11^ and MCM-41^12^), showing the potential applications of MIL-101-OH for adsorptive removal of naproxen from water.

Before evaluation of reusability of the MIL-101-OH, the stability of the MOF, after naproxen adsorption, was checked using XRD and SEM. As shown in Supplementary Figure 2, there is little change of XRD patterns of MIL-101 after modification to introduce –OH and after naproxen adsorption. Moreover, SEM images of Supplementary Figure 3 showed similar results. The energy dispersive X-ray spectroscopy (EDX) results (Supplementary Table 2) show that the chemical composition of MIL-101 did not change much with modification to introduce –OH and after naproxen adsorption. However, the contents of C and Cr of the MOF increased and decreased slightly, respectively, upon modification and adsorption. The EDX analysis results are understandable because of introduction of carbonaceous materials on the MOF by modification and adsorption of naproxen. All of these results confirm the stability of the MIL-101-OH in adsorption of naproxen, suggesting the possible reusability in adsorptions.

**Figure 6:**
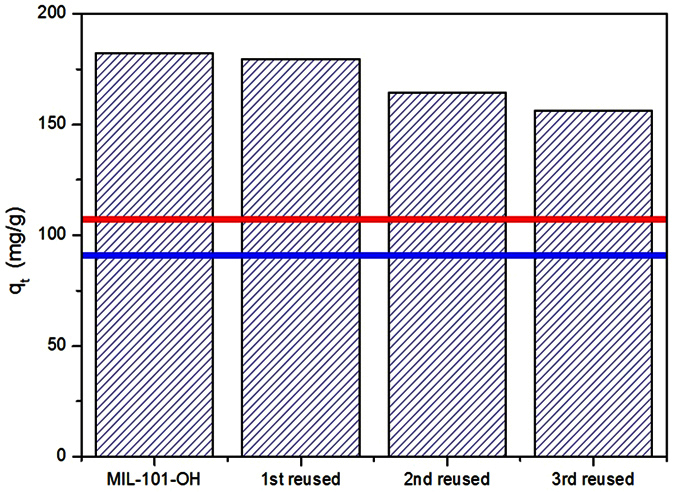
Effect of recycle numbers on the adsorbed amount of naproxen over MIL-101-OH. The upper and lower horizontal lines show the adsorbed amount of naproxen over pristine MIL-101 and activated carbon, respectively (the adsorption time: 12 h and initial concentration of naproxen: 50 ppm).

**Figure 7:**
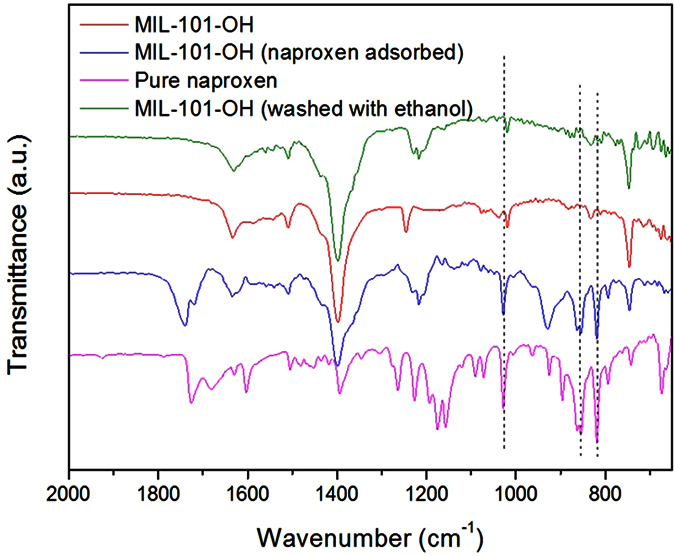
FTIR spectra of naproxen, MIL-101-OH, MIL-101-OH (with adsorbed naproxen), and purified MIL-101 (by ethanol washing of the adsorbed MIL-101).

Reusability of adsorbents is very important for their possible application in commercial plants. As it showed the highest adsorption capacity per unit weight, MIL-101-OH was washed with ethanol, a common solvent, and used again for the adsorptive removal of naproxen from water. As shown in Fig. 6, the performances of MIL-101-OH decreased slightly with increasing the number of recycles. However, the performance after the third cycle was still much higher than that of activated carbon or virgin MIL-101 (highlighted with colored horizontal lines in the figure), showing the competitiveness of MIL-101-OH for naproxen adsorption. FTIR spectra showed not only adsorbed naproxen and successful removal with solvent washing, based on the bands corresponding to naproxen (Fig. 7).

Even though MIL-101 does not contain toxic Cr(VI) ions, one may suspect the practical applications of MIL-101 containing Cr(III), with or without modifications, in water purifications. The results of this study, however, can suggest the possible applications of suitable MOFs in water purification via adsorption, considering synthesis of highly porous MOFs composed of non-toxic metal ions such as iron, aluminum, titanium, alkali metals and alkaline earth metals.

## Discussion

Understanding the possible mechanism of adsorption is important for efficient removal and separation of chemicals. Naproxen, the adsorbate, has polar functional groups such as carboxylic acid and ether. Of the studied adsorbents, the MIL-101s with –OH and –NH_2_ groups were effective for naproxen adsorption, while MIL-101-NO_2_ was very poor at adsorption. Therefore, MIL-101 was very efficient at adsorbing naproxen when the MOF was functionalized with hydroxyl (or amino) groups with hydrogen atoms that can be used as H-donors in H-bonding; however, MIL-101 functionalized with H-acceptors was not efficient for naproxen adsorption. Moreover, MIL-101-(OH)_2_ having two H-donors was very effective to show the highest adsorption capacity based on surface area (Table 1 and Figs 3b and 4b). Therefore, H-bonding is a possible mechanism to explain the adsorption of naproxen by functionalized MIL-101s. Moreover, the almost identical adsorption capacities of MIL-101 and MIL-101-OH at pH 10 suggests that the amounts of naproxen adsorbed might be the same if there was no contribution from H-bonding (i.e., at a pH high enough for complete deprotonation of MIL-101-OH). Considering the pH of the solution (5.4) and the pKa of naproxen (~4.2), the very poor performance of MIL-101-NO_2_ might be explained by the negligible contribution of H-bonding (because of the absence of H-donors in naproxen). In fact, the adsorptive performances of MIL-101 and MIL-101-NO_2_ per unit surface area were very similar to each other (Figs 3b and 4b).

**Figure 8:**
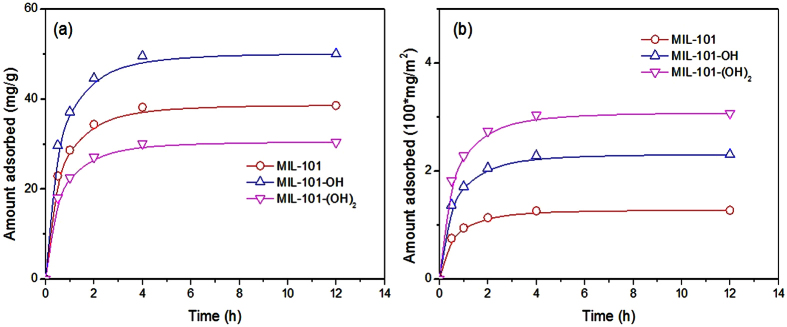
Effect of adsorption times on the adsorbed amounts of ibuprofen over MIL-101s. (**a**,**b**) Show the adsorbed amounts of ibuprofen based on the unit weight and surface area, respectively, of adsorbents. The initial concentration of ibuprofen was 50 ppm. The legends in (**b**) are the very same as those in (**a**).

**Figure 9:**
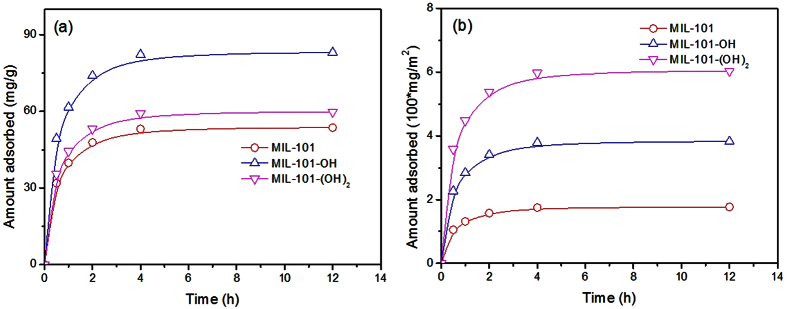
Effect of adsorption times on the adsorbed amounts of oxybenzone over MIL-101s. (**a**,**b**) Show the adsorbed amounts of oxybenzone based on the unit weight and surface area, respectively, of adsorbents. The initial concentration of oxybenzone was 50 ppm. The legends in (**b**) are the very same as those in (**a**).

In order to confirm the mechanism of H-bonding, similar PPCPs such as ibuprofen and oxybenzone were adsorbed over MIL-101 with or without free hydroxyl groups (MIL-101, MIL-101-OH and MIL-101-(OH)_2_). The MIL-101-OH showed the highest adsorption capacities (Figs 8a and 9a) for the ibuprofen and oxybenzone (per unit weight) even though the surface area of the MOF was not the highest. This result is very similar to the naproxen adsorption as shown in Figs 3a and 4a. Figures 8b and 9b show that the amounts of adsorbed ibuprofen and oxybenzone (per unit surface area) decrease on the order MIL-101-(OH)_2_ > MIL-101-OH > MIL-101, which is very similar to the tendencies in Figs 3b and 4b. The relative adsorbed amounts of the three PPCPs over the three MOFs (after 12 h (q_12h_); where, the value of the MIL-101 was set as 100) were shown in Table 3. Irrespective of the PPCPs, the q_12h_ decreases on the order MIL-101-(OH)_2_ > MIL-101-OH > MIL-101. Interestingly, the degree of increase of q_12h_ is naproxen > oxybenzone > ibuprofen (for example, the q_12h_ values of MIL-101-(OH)_2_ for naproxen, oxybenzone and ibuprofen are 3.97, 3.41 and 2.41 times of MIL-101, respectively)). This interesting tendency can be explained with the number and status of oxygen in PPCPs if H-bonding is one of the important mechanisms of adsorption. The functional groups (containing oxygen) of naproxen, oxybenzone and ibuprofen are also summarized in Table 3. The lowest increases of q_12h_ of ibuprofen with number of –OH on MOFs can be explained with the lowest number (2 ea) of oxygen in the PPCP. The higher increases of q_12h_ of naproxen (compared with oxybenzone) with number of –OH on MOFs might be because of O^−^ (consequently, strong H-bond with HO of MIL-101s) from –COOH under the adsorption condition (pH: 5.4). Therefore, the results with naproxen, ibuprofen and oxybenzone adsorptions can be explained clearly with favorable H-bonding between H of HO (on MOFs) and O of adsorbates (PPCPs). The plausible mechanism for adsorption of the naproxen over MIL-101-OH can be represented as Supplementary Scheme 1, where, the H-bond was drawn with dotted line. Mechanism for the adsorption of other PPCPs over the MOFs can be similarly presented.

Very recently, a similar adsorption mechanism was reported^15^ for adsorption of naproxen by bone char. Moreover, H-bonding has been an important interaction mechanism for explaining various processes including adsorption by MOFs^59^,^60^,^61^,^62^,^63^,^64^,^65^. As suggested earlier^47^, acid-base interactions may also explain the adsorption of naproxen by basic MIL-101-NH_2_. The experimental results also showed the importance of the surface area (Figs 3 and 4) of MIL-101s and the pH (Fig. 5) of solutions for naproxen adsorption, suggesting contributions by conventional van der Waals force and electrostatic interactions, respectively. The latter mechanism has already been suggested in previous works using both pristine and functionalized MIL-101^46^,^47^. The former mechanism has been reported in various adsorptions where there was no other special/strong interaction mechanism^30^,^66^.

In conclusion, a typical MOF with high porosity (MIL-101) was modified to introduce several functional groups such as –OH, –(OH)_2_, –NH_2_, and –NO_2_ in order to use it for the adsorptive removal of PPCPs such as naproxen, ibuprofen, and oxybenzone from an aqueous solution. Even though the surface area of the virgin MOF decreased noticeably, some of the modified MIL-101s were very effective at the PPCPs adsorption. MIL-101-OH and MIL-101-(OH)_2_ showed the highest PPCPs uptakes based on weight and surface area, respectively. From the adsorption results of naproxen (and of similar PPCPs such as ibuprofen and oxybenzone), H-bonding was suggested to be an important mechanism for explaining the enhanced efficiency of the MIL-101s with H-donor functionalities (MIL-101–OH, MIL-101-(OH)_2_, and MIL-101–NH_2_). Finally, MIL-101-OH is suggested to be a potential commercial adsorbent for PPCPs removal based on its reusability and competitive adsorption when compared with carbonaceous materials, mesoporous materials, and pristine MIL-101.

## Methods

### Chemicals and synthesis/modification of the adsorbents

Reagents and solvents were commercially available products and used without any further purification. Chromium nitrate nonahydrate (Cr(NO_3_)_3_∙9H_2_O, 99%) and terephthalic acid (TPA, 99%) were purchased from Samchun and Junsei Chemicals, respectively. Ethanolamine (ETA, 98%) and diethanolamine (DEA, 99%) were obtained from Alfa Aesar. Ethanol (99.5%), nitric acid (60%), sulfuric acid (98%), and toluene (99.5%) were procured from OCI chemicals. Naproxen (98%) and tin chloride (SnCl_2_, 98%) were obtained from Sigma-Aldrich. Ibuprofen (99%) and oxybenzone (98%) were procured from Alfa Aesar.

**Figure 10:**
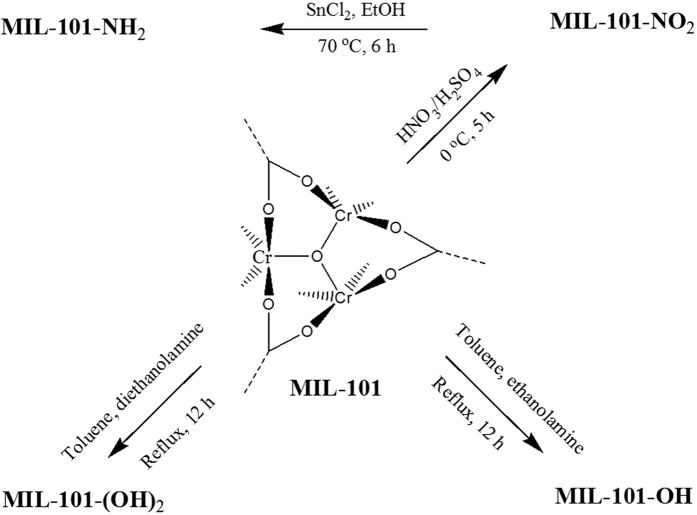
A scheme to show the modification methods to introduce various functional groups on MIL-101.

MIL-101 was synthesized from Cr(NO_3_)_3_∙9H_2_O, TPA, and deionized water in a similar manner to a previously described method^51^,^67^. The –OH functionalized MIL-101s (named MIL-101-OH and MIL-101-(OH)_2_) were synthesized via grafting utilizing reported procedures^28^,^47^. The dehydrated MIL-101 (0.3 g) was suspended in anhydrous toluene (30 mL) in a round-bottomed flask equipped with a reflux condenser and a magnetic stirrer, and each of 1 mmol of ETA (or DEA) was added to this suspension. The mixture was continuously stirred and refluxed for 12 h. The obtained solid was cooled to room temperature, separated, washed with ethanol/de-ionized water, and dried at room temperature. MIL-101-NO_2_ was obtained by nitration of MIL-101 following a method reported earlier^68^. The nitration of dehydrated MIL-101 (0.3 g) was done at 0 °C (under ice cooling) for 5 h by using 50 mL of a mixture of acids (nitric acid (0.1 M) and sulfuric acid (0.1 M)). The product was separated, washed with ethanol/de-ionized water, and dried at room temperature. To obtain MIL-101-NH_2_, the MIL-101-NO_2_ was reduced at 70 °C for 6 h using SnCl_2_∙2H_2_O in ethanol, which is a previously known process^68^. The procedures for the functionalization of MIL-101 are summarized in Fig. 10.

### Characterization

X-ray powder diffraction (XRD) patterns of MIL-101s were obtained with a D2 Phaser diffractometer (Bruker, with CuKα radiation). FTIR spectra were acquired with a Jasco FTIR-4100 (ATR, maximum resolution: 0.9 cm^−1^). Nitrogen adsorptions were measured at −196 °C with a surface area/porosity analyzer (Micromeritics, Tristar II 3020) after evacuation at 150 °C for 12 h. The surface areas of adsorbents were calculated using the BET equation.

### General procedures for the adsorption experiments

Naproxen solutions of the desired concentrations were prepared by dissolving naproxen in deionized water/acetone (99:1 v/v). Naproxen concentrations were determined by measuring the absorbance of the solutions at 230 nm using a spectrophotometer (Shimadzu UV spectrophotometer, UV-1800). A calibration curve for naproxen was obtained from the spectra of the standard naproxen (1–10 ppm) solution.

Before adsorption, the adsorbents were dried overnight under vacuum at 100 °C and stored in a desiccator. An exact amount of the adsorbents (5.0 mg) was put in a naproxen solution (48 mL, pH = 5.4) with a fixed concentration. The naproxen solution containing the adsorbents was mixed well with magnetic stirring for a fixed time (30 min to 12 h) at 25 °C. After adsorption for a pre-determined time, the solution was separated from the adsorbents with a syringe filter (PTFE, hydrophobic, 0.5 μm), and the naproxen concentration was calculated from its absorbance from the UV spectra. If needed, a UV measurement was conducted after diluting the naproxen solution. To measure the adsorbed amount of naproxen at various acidities, the pH of the naproxen solution was adjusted with 0.1 M aqueous solutions of HCl or NaOH.

A mass-balance relationship, Eq. (1), was applied to calculate the amount of naproxen adsorbed onto different adsorbents under various conditions:


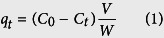


where *C*_0_ and *C*_t_ (mg/L) are the liquid-phase concentrations of naproxen at time = 0 and *t*, respectively, and *V* (L) and *W* (g) are the volume of the solution and the weight of the adsorbent, respectively. Adsorption of ibuprofen and oxybenzone was done similarly for 0.5–12 h.

Adsorption isotherms of naproxen were obtained after adsorption for 12 h. A Langmuir isotherm was used to calculate the maximum adsorption capacity of each adsorbent, and the linear form of Langmuir isotherm equation is given as^69^,^70^:


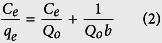


where *C*_e_ (mg/L) is the equilibrium concentration of the adsorbate, *q*_e_ (mg/g) is the amount of adsorbate adsorbed, and *Q*_0_ (mg/g) is the Langmuir constant (or maximum adsorption capacity). Therefore, *Q*_0_ can be obtained from the reciprocal of the slope of a plot of *C*_e_/*q*_e_ against *C*_e_.

Regeneration of used adsorbent was carried out at room temperature by mixing the used adsorbent and ethanol for 4 h under magnetic stirring, followed by sonication for 1 h, filtration, washing with ethanol, and finally drying in a vacuum oven for further use. A similar regeneration process was repeated up to the third recycle.

## Additional Information

**How to cite this article**: Seo, P. W. *et al*. Adsorptive Removal of Pharmaceuticals and Personal Care Products from Water with Functionalized Metal-organic Frameworks: Remarkable Adsorbents with Hydrogen-bonding Abilities. *Sci. Rep.* **6**, 34462; doi: 10.1038/srep34462 (2016).

## Supplementary Material

Supplementary Information

## Figures and Tables

**Figure 1:**
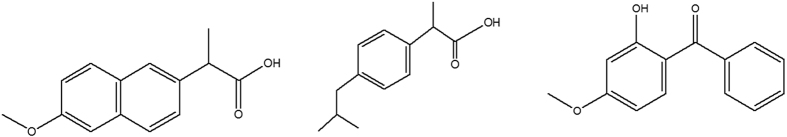
Chemical structure of naproxen, ibuprofen and oxybenzone.

**Table 1:** BET surface areas and maximum adsorption capacities (based on weight and surface area of adsorbent) of MIL-101s for naproxen.

Adsorbent	SA_BET_ (m^2^/g)	Q_0_ (mg/g)	Q_0_ (100*mg/m^2^)	r^2^
MIL-101	3030	114	3.76	0.983
MIL-101-OH	2170	185	8.52	0.991
MIL-101-(OH)_2_	990	136	13.7	0.989
MIL-101-NH_2_	1892	147	7.77	0.988
MIL-101-NO_2_	1620	66.1	4.08	0.986

**Table 2:** Maximum adsorption capacities (Q_0_) or adsorbed amount after 24 h at equilibration (q_24 h_) of various adsorbents for naproxen.

Adsorbent	Q_0_ or q_24 h_ (mg/g)	Reference
Bone char	<5.0	15
AC	106	56
AC	18.9	57
AC	88	58
AC	81	48
AC	159	12
SBA-15	0.16*	11
Cu-NH_2_-g-SBA-15	0.65*	11
Ni-NH_2_-g-MCM-41	3.9*	12
MCM-41	2.8*	12
MIL-101	114	This work
MIL-101-OH	185	This work

^*^Mean adsorbed amounts after 24 h of equilibration (q_24 h_).

**Table 3:** Relative adsorbed amounts of three PPCPs over the three MOFs (MIL-101, MIL-101-OH and MIL-101-(OH)_2_) after 12 h of adsorption (q_12 h_).

PPCP (number/status of O species)	Functional group of PPCP*	MIL-101	MIL-101-OH	MIL-101-(OH)_2_
Naproxen (2O, 1O^−^)	-COO^−^, -O-	100	238	397
Ibuprofen (1O, 1O^−^)	-COO^−^	100	181	241
Oxybenzone (3O)	-OH, =O, -O-	100	216	341

^*^At the condition of adsorption (pH: 5.4).

The q_12 h_ values are based on unit surface area and the q_12 h_ of MIL-101 was set 100 to check easily the effect of –OH of MOFs on the adsorbed amounts.
