# Supplementary material for: Adsorptive Removal of Pharmaceuticals and Personal Care Products from Water with Functionalized Metal-organic Frameworks: Remarkable Adsorbents with Hydrogen-bonding Abilities
Source: Sci Rep. 2016 Oct 3;6:34462. doi: 10.1038/srep34462 (PMC5046089; doi:10.1038/srep34462)
Supplement: Supplementary Information [file srep34462-s1.doc]

<Supplementary Information>

**Adsorptive Removal of Pharmaceuticals and Personal Care Products from Water with Functionalized Metal-organic Frameworks: Remarkable Adsorbents with Hydrogen-bonding Abilities**

Pill Won Seo, Biswa Nath Bhadra, Imteaz Ahmed, Nazmul Abedin Khan, and Sung Hwa Jhung^*^

Department of Chemistry, Kyungpook National University, Daegu 702-701, Korea, Fax: (+) 82-53-950-6330; E-mail: [sung@knu.ac.kr](mailto:sung@knu.ac.kr)

*Corresponding Author: Prof. Sung Hwa Jhung

Fax: 82-53-950-6330/Tel: 82-53-950-5341

Email: sung@knu.ac.kr

Supplementary Table 1. The physical properties of the PPCPs used in this study.

| PPCPs | Molar mass (g/mol) | pKa | Log (Kow)  (Kow or Pow: The octanol-water partition coefficient) |
| --- | --- | --- | --- |
| naproxen | 230.3 | 4.2 [1] | 3.18 [2] |
| ibuprofen | 206.3 | 4.6 [1] | 3.97 [2] |
| oxybenzone | 228.3 | 7.6 [1] | 3.8 [3] |

References:

[1] N. Fontanals, P. A.G. Cormack, D. C. Sherrington, R. M. Marce, F. Borrull, *J. Chromatogr. A*, 1217, 2855–2861, 2010 .

[2] N. Ni, T. Sanghvi, S. H. Yalkowsky, *Pharmaceutical Research*, 19, 1862-1866, 2002.

[3] S. Kasichayanula, J. D. House, T. Wang, X. Gu, *Toxicology Applied Pharmacology* 223, 187–194, 2007.

Supplementary Table 2. Compositions (based on SEM-EDX) of MIL-101 and MIL-101-OH (fresh and after naproxen adsorption).

| Adsorbent | C content (wt %) | O content (wt %) | Cr content (wt %) |
| --- | --- | --- | --- |
| MIL-101 | 48.6 | 32.6 | 18.9 |
| MIL-101-OH | 50.9 | 32.0 | 17.2 |
| MIL-101-OH (after adsorption of naproxen) | 53.1 | 32.5 | 14.4 |

Supplementary Scheme 1. Plausible adsorption mechanism of naproxen over MIL-101-OH via H-bonding (represented as dotted lines).

Supplementary Figure 1. Langmuir plots for naproxen adsorption over MIL-101s. Figures (a) and (b) show the Langmuir plots based on the unit weight and surface area, respectively, of adsorbents.

Supplementary Figure 2. XRD patterns of MIL-101 and MIL-101-OH (fresh and after naproxen adsorption).


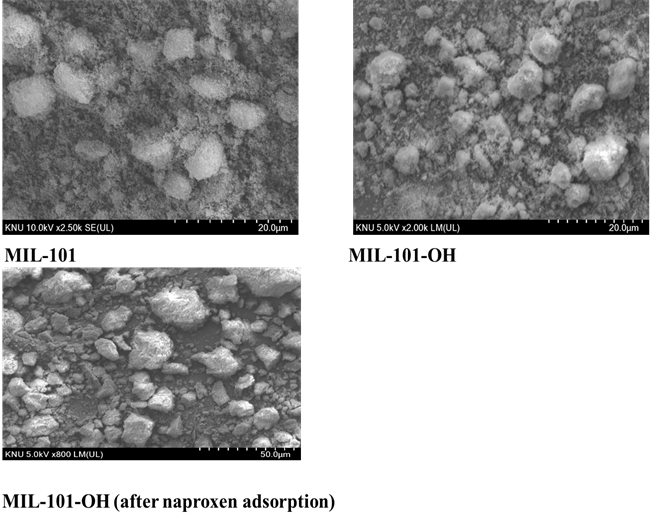


Supplementary Figure 3. SEM images of MIL-101 and MIL-101-OH (fresh and after naproxen adsorption).
